# The Exchange Mechanism of Alkaline and Alkaline-Earth Ions in Zeolite N

**DOI:** 10.3390/molecules24203652

**Published:** 2019-10-10

**Authors:** Monireh Khosravi, Vinuthaa Murthy, Ian D R Mackinnon

**Affiliations:** 1 Institute for Future Environments and Science and Engineering Faculty, Queensland University of Technology, Brisbane QLD 4001, Australia; ian.mackinnon@qut.edu.au; 2 College of Engineering, IT and Environment, Charles Darwin University, Darwin NT 0909, Australia; vinuthaa.murthy@cdu.edu.au

**Keywords:** zeolite N, ion exchange mechanism, diffusion, molecular dynamics, ammonium, monovalent cation, divalent cation, concentration profile, self-diffusion coefficient, radial distribution function

## Abstract

Zeolite N is a synthetic zeolite of the EDI framework family from the more than 200 known zeolite types. Previous experimental laboratory and field data show that zeolite N has a high capacity for exchange of ions. Computational modelling and simulation techniques are effective tools that help explain the atomic-scale behaviour of zeolites under different processing conditions and allow comparison with experiment. In this study, the ion exchange behaviour of synthetic zeolite N in an aqueous environment is investigated by molecular dynamics simulations. The exchange mechanism of K^+^ extra-framework cations with alkaline and alkaline-earth cations NH_4_^+^, Li^+^, Na^+^, Rb^+^, Cs^+^, Mg^2+^ and Ca^2+^ is explored in different crystallographic directions inside the zeolite N structure. Moreover, the effect of different framework partial charges on MD simulation results obtained from different DFT calculations are examined. The results show that the diffusion and exchange of cations in zeolite N are affected by shape and size of channels controlling the ion exchange flow as well as the nature of cation, ionic size and charge density.

## 1. Introduction

The potassium-rich zeolite K-F(Cl), later renamed zeolite N with the general formula K_12_Al_10_Si_10_O_40_Cl_2_.5H_2_O, was initially synthesised by Barrer et al. in 1953 [1]. Christensen and Fjellvag determined the crystal structure of zeolite N using high resolution X-ray and neutron diffraction data [2,3]. The structure of zeolite N is orthorhombic with space group I222 and lattice parameters a = 9.9041 Å, b = 9.8860 Å and c = 13.0900 Å. Zeolite N is in the EDI framework group and is considered a fibrous zeolite. The Si/Al ratio of the end-member zeolite N is one and, in general, affords a high ion-exchange capacity. The framework of zeolite N has low tortuosity and the predominant eight membered channel along the *c* axis provides an unimpeded path for ions to transfer or transport to exchangeable sites inside the cages. Furthermore, the extra-framework potassium cations can be exchanged due to their accessible positions and weak electrostatic bonds to water molecules and framework atoms. These properties make zeolite N an interesting candidate for ion-exchange applications.

The high capacity of zeolite N for selective ion exchange applications, compared with competitive natural zeolites, has been verified by experimental studies. Mackinnon et al. [4] and Thornton et al. [5] indicate that zeolite N has a robust potential for ammonium removal (up to 90%) from return side streams of wastewater treatment plants with an inlet ammonium concentration ranging between 600 mg/L and 900 mg/L. These investigations reported 45–55g NH_4_^+^-Nkg^−1^ ammonium loading capacity for zeolite N, while the natural zeolite, clinoptilolite, used extensively for ammonium removal applications, shows a much lower loading capacity for ammonium, in the range of 0.94–21.52g NH_4_^+^-Nkg^−1^ [5,6]. In agronomy, Zwingmann et al. [7] demonstrated that adding small amounts (0.4%) of zeolite N to sandy soils effectively increased NH_4_^+^ retention capability. In controlled glasshouse trials, Zwingmann et al. [7] showed that the exchange performance of zeolite N is 11 times higher than natural zeolite clinoptilolite under the same conditions. In other trials, Zwingmann et al. [8] showed that K from zeolite N is available for plants and is released by cation exchange, particularly when NH_4_^+^, Ca^2+^ and Mg^2+^ are present in a nutrient solution.

Moreover, the exchange behaviour of zeolite N is investigated under different experimental conditions. Mackinnon et al. [9] and Thornton et al. [6] show that the initial solution concentration and pH impact the ammonium uptake by zeolite N. They reported that increasing the ammonium concentration in solution results in an increase in the rate and capacity of ammonium removal from solution. Thornton et al. [6] found that pH 6–7 is the optimum pH for ammonium removal. Thornton et al. [6] showed that the capacity of zeolite N for ammonium uptake decreases by 30% in the presence of competing cations, sodium, calcium and magnesium. Mackinnon et al. [9] reported that the presence of magnesium and calcium in a mixed solution (with different cation concentration compared to the Thornton et al. study [6]) has no significant effect on ammonium uptake. However, the presence of sodium slightly decreases the capacity for removal of ammonium. These experimental studies indicate a preference by zeolite N for univalent cation selectivity compared to divalent cations.

These experimental studies demonstrate the high ion exchange capacity and exchange isotherms of zeolite N. However, these experiments are not able to explain the exchange and diffusion mechanism of cations within the complex porous structure of zeolite N. In our recent study [10] we simulated the exchange of univalent cations, NH_4_^+^, Na^+^, K^+^, Rb^+^ and Cs^+^, in a zeolite N membrane using molecular dynamics calculations. We studied the structural and dynamic behaviour of ions inside a zeolite N membrane. The results show that zeolite N prefers K^+^ exchange with NH_4_^+^ rather than with Na^+^, Rb^+^ or Cs^+^. Moreover, the behaviour of zeolite N at different hydration levels was investigated. The outcomes of our molecular dynamics calculations are in good agreement with experimental data for ammonium and sodium exchange with potassium in hydrated zeolite N. This modelling approach and outcomes that, in general, conform with experimental data, show that computational modelling can be used to understand detailed, atomic scale mechanistic interactions for ion exchange of zeolite N.

In this study, we present further details of ion exchange mechanisms for zeolite N based on exchange of monovalent cations inside a zeolite N membrane, as well as the relative performance of Li^+^, Ca^2+^ and Mg^2+^ cations. We explore ion retention within a zeolite N membrane along different crystallographic directions, [001] and [110], as well as the site preference of exchanged cations in the zeolite N structure. For our earlier study [10], we utilised partial charges for framework atoms (i.e., Si and Al) calculated by Salmas et al. [11] for zeolite LTA as a guide. However, our recent study [12] shows that the partial charges of framework Si and Al atoms can be substantially different depending on the functional used in DFT calculations and on the zeolite type. Consequently, we utilise optimised values for Si and Al partial charges identified in [12] and consider the effect of these different partial charges for framework Si and Al atoms on the dynamic behaviour of zeolite N. 

## 2. Results

The statistics, structural and dynamics results of ions and water molecules from MD simulations are presented in this section. We compare results obtained for different K^+^/M^n+^ systems in the ZM-001 and ZM-110 membranes. These designations for each membrane, ZM-001 and ZM-110, refer to the specific crystallographic orientations simulated in this study (see Methods).

### 2.1. Ion Retention

**Figure 1:**
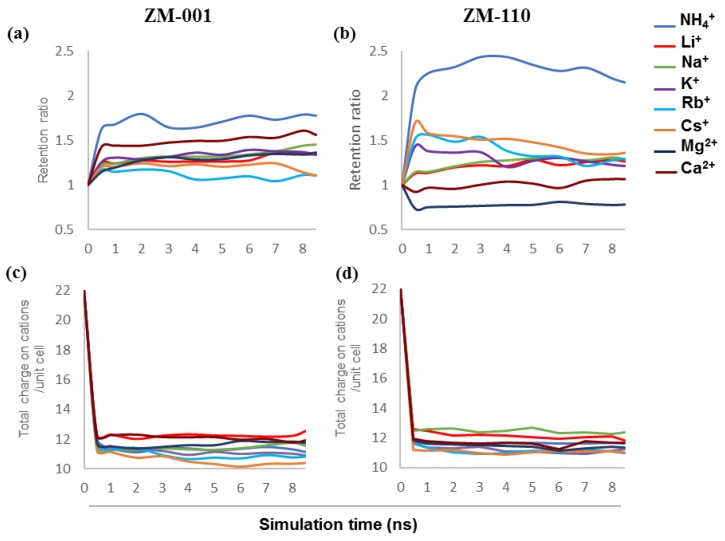
The retention ratio of guest to host ions in (**a**) ZM-001 and (**b**) ZM-110, and the total charge on cations per unit cell of (**c**) ZM-001 and (**d**) ZM-110.

The retention of ions and water molecules inside ZM-001 and ZM-110 over 8.5 ns simulation times are plotted in Figure 1a,b. These plots show the retention ratio between guest cations and extra-framework K^+^ ions of zeolite N membranes for ZM-001 and ZM-110. The retention ratio of guest to host ions in ZM-001 is higher than that for all exchanging systems. However, ZM-110 shows different behaviour for the retention of ions. NH_4_^+^ has the highest retention ratio in both membranes. The ammonium to K^+^ retention ratio in ZM-001 fluctuates around 1.7 over time. In ZM-110, the ammonium retention ratio increases up to 2.5 and after 4 ns decreases, but remains higher than ZM-001. The Li^+^ and Na^+^ ions show roughly the same behaviour in both membranes with slightly higher retention ratios over time in ZM-001. Both types of membranes initially release Li^+^ and Na^+^ to the solution but overtime, especially after 6 ns in ZM-001, the membrane exchanges more K^+^ with Li^+^ and Na^+^ in the solution. The K^+^ retention equilibrates in ZM-001 faster than for ZM-110. The retention ratios for Rb^+^ and Cs^+^ in ZM-001 are lower than ZM-110; however, the retention ratios decrease over time in ZM-110. The retention ratios for Mg^2+^ and Ca^2+^ are higher in ZM-001 compared to ZM-110. The retention ratio of Mg^2+^ and Ca^2+^ in ZM-110 are about one and below one, respectively and much lower than other ions studied.

Experimentally, the zeolite N unit cell contains 12 monovalent cations compensating the negative charge of framework (−10e) and two chloride ions [2]. We find that the average total compensating charge on cations per unit cell of ZM-001 and ZM-110 (Figure 1c,d) in most exchanging systems are less than 12 except for the Li^+^ system in ZM-001 and Li^+^ and Na^+^ exchanging systems in ZM-110. The retention results show that the membranes retain fewer cations compared to the original zeolite N membranes without guest cations (K-ZM-001 and K-ZM-110). Here, we consider the total compensating charge on the cations rather than the number of cations (Figure 1c,d). Investigating the average number of Cl^−^ ions retained in each unit cell of the membranes (Figure S1c,d) shows that the number of chloride ions decreases over time especially in ZM-001. In the K^+^/Li^+^ system of ZM-110, the membrane adsorbs more than 2 Cl^−^ anions per unit cell at the initial stages of ion exchange but subsequently releases excess Cl^−^ ions into solution. 

Zeolite N contains eight water molecules per cage [10]. Counting the average number of water molecules in each cage of ZM-110 (Figure S1e,f) demonstrates that the membrane adsorbs water molecules in K^+^/Li^+^, K^+^/Na^+^, K^+^/K^+^, K^+^/Rb^+^, K^+^/Mg^2+^ and K^+^/Ca^2+^ systems up to one extra water molecule per cage but releases water molecules in K^+^/NH_4_^+^ and K^+^/Cs^2+^ systems (Figure S1f). However, in the ZM-001 membrane only K^+^/Li^+^ and Mg^2+^/K^+^ systems adsorb water molecules (Figure S1e). Zeolitic water molecules in K^+^/NH_4_^+^, K^+^/Na^+^, K^+^/K^+^, K^+^/Rb^+^, K^+^/Cs^+^ and K^+^/Ca^2+^ systems exit the ZM-001 membrane within 1 ns of MD simulations due to the stress created by addition of excess cations inside the membrane. Over time, the membrane re-adsorbs water molecules to reach an equilibrium condition. This feature is most obvious for the K^+^/Cs^+^ system. 

Table 1 presents the number and percentage of retained extra-framework K and Cl ions, water molecules and guest ions inside ZM-001 and ZM-110 after 8.5 ns simulation time. As shown in Table 1 in the K^+^/K^+^ systems of ZM-001 and ZM-110, ~50% of the potassium is retained in the membrane. The number of potassium ions exchanged by both membranes in the K^+^/K^+^ systems after 8.5 ns simulation is similar to that in the K-ZM-001 and K-ZM-110 membrane without guest cations. Ca^2+^ and NH_4_^+^ ions have the highest retention in the ZM-001 membrane with 67.5% and 66.2% retention respectively. The Li^+^, Na^+^ and Mg^2+^ show more than 60% retention in ZM-001 (with 63.7%, 63.2% and 62.5% retention, respectively). However, Rb^+^ and Cs^+^, with 51.4% and 50.1% retention show the lowest capacity to remain in the ZM-001 membrane. In the ZM-110 membrane, NH_4_^+^ shows the highest retention of 70.8% while Na^+^, Li^+^, Cs^+^, Rb^+^ and Ca^2+^ show more than 50% retention of 64.3%, 59.4%, 58.9%, 56.7% and 54.9%, respectively. Mg^2+^ shows the lowest retention of 44.8%.

In general, zeolite N contains two different exchange sites, SI and SII, which when occupied by extra-framework potassium are designated K1 and K2 [2]. The K-ZM-001 and K-ZM-110 membranes without guest cations contain 32 K1 and 64 K2 cations. We estimate the number of ions in K1 and K2 sites in order to validate their relative exchange capability. The retention of K1 and K2 in ZM-001 and ZM-110 shows different behaviour depending on the guest ions. In K^+^/NH_4_^+^, K^+^/Na^+^ and K^+^/Cs^+^ systems, the percentage of K1 retained in the membrane is higher compared to K2. However, in K^+^/Li^+^ and K^+^/K^+^ systems of both membranes K2 retention percentage is higher than K1. In the K^+^/Rb^+^ system, the K2 retention percentage is more than K1 in ZM-001 and less than K1 in ZM-110. However, K1 and K2 retention in K^+^/Mg^2+^ and K^+^/Ca^2+^ systems of both membranes show the reverse behaviour to the K^+^/Rb^+^ system.

### 2.2. Ion Distribution

MD calculations allow determination of the relative distribution of ions and bonding characteristics between different atom pairs within the membranes and in the surrounding electrolytes. The proportion of host and guest ions inside and outside the membranes are determined by concentration profiles. The location of ions inside the channels and cages of membranes are estimated with ion density field maps. The structural arrangements of host and guest ions around framework atoms are characterised by calculating the radial distribution functions (RDFs).

**Figure 2:**
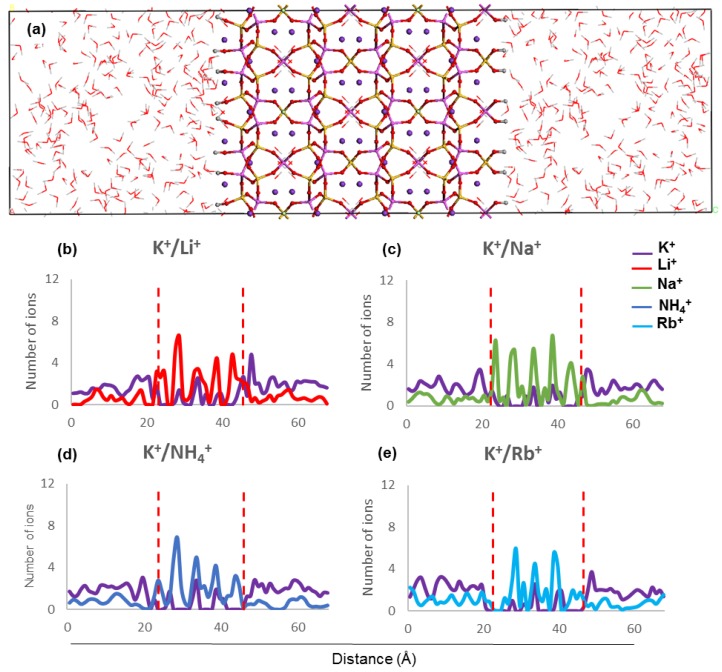
(**a**) ZM-001 simulation box along the *z* direction and (**b**–**e**) ion concentration profiles along the *z* direction after 8.5 ns MD simulations. The two red dashed lines indicate the location of ZM-001 surfaces in the electrolyte solution.

**Figure 3:**
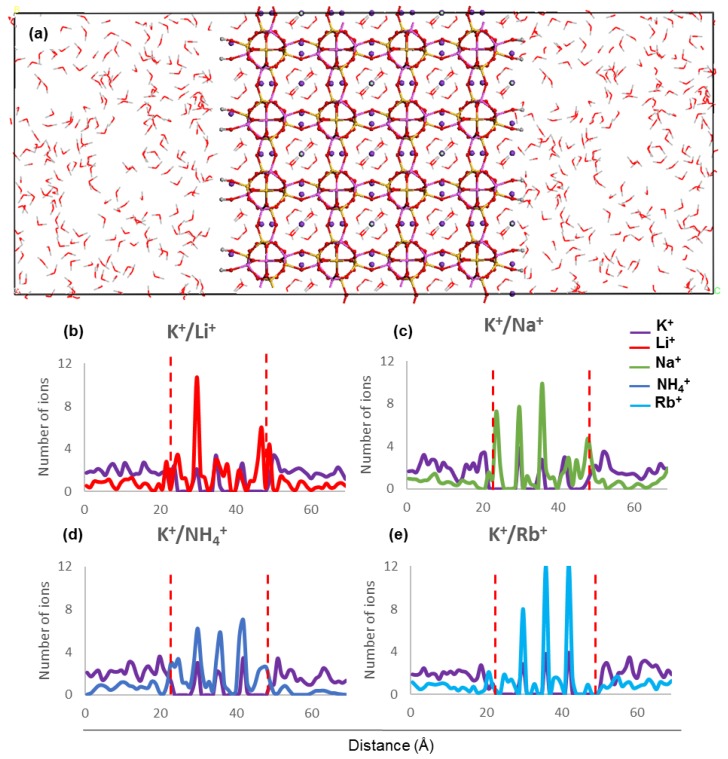
(**a**) ZM-110 simulation box along the *z* direction and (**b**–**e**) ion concentration profiles along the *z* direction after 8.5 ns MD simulations. The two red dashed lines indicate the location of ZM-110 surfaces in the electrolyte solution.

The concentration profiles of host and guest cations along the *z* direction inside ZM-001 and ZM-110 and their surrounding solution after 8.5 ns MD simulation are presented in Figure 2 and Figure 3, respectively (as well as Figures S2 and S3). Ion concentration profiles show the occupation of middle parts of the membrane by NH_4_^+^, Li^+^, Na^+^, K^+^, Rb^+^, Cs^+^, Mg^2+^ and Ca^2+^. The distribution of these ions in the middle of each membrane is uniform for NH_4_^+^, K^+^, Rb^+^ and Cs^+^ cations. However, Li^+^, Na^+^, Mg^2+^ and Ca^2+^ cations are unevenly distributed in the middle of the membranes. The opening of channels in both membranes are occupied by guest ions in K^+^/NH_4_^+^, K^+^/Li^+^, K^+^/Na^+^, K^+^/Mg^2+^ and K^+^/Ca^2+^ systems. However, the opening of channels are devoid of any cations in the K^+^/K^+^, K^+^/Rb^+^ and K^+^/Cs^+^ systems of both membranes. Moreover, concentration profiles show the adsorption of Li^+^ and Mg^2+^ guest ions on the surfaces of ZM-110. In all systems of both membranes, K^+^ cations prefer to stay in the middle of the membrane and the concentration of K^+^ ions in the solution is higher near membrane surfaces.

**Figure 4:**
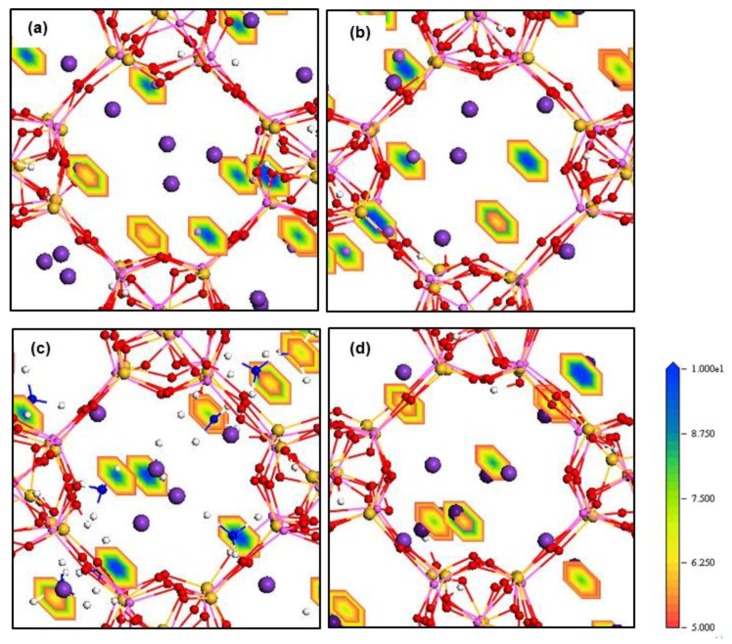
Density field maps of M^n+^ guest cations in (**a**) K^+^/Li^+^, (**b**) K^+^/Na^+^, (**c**) K^+^/NH_4_^+^ and (**d**) K^+^/Cs^+^ systems retained inside ZM-001 after 8.5 ns MD simulations.

**Figure 5:**
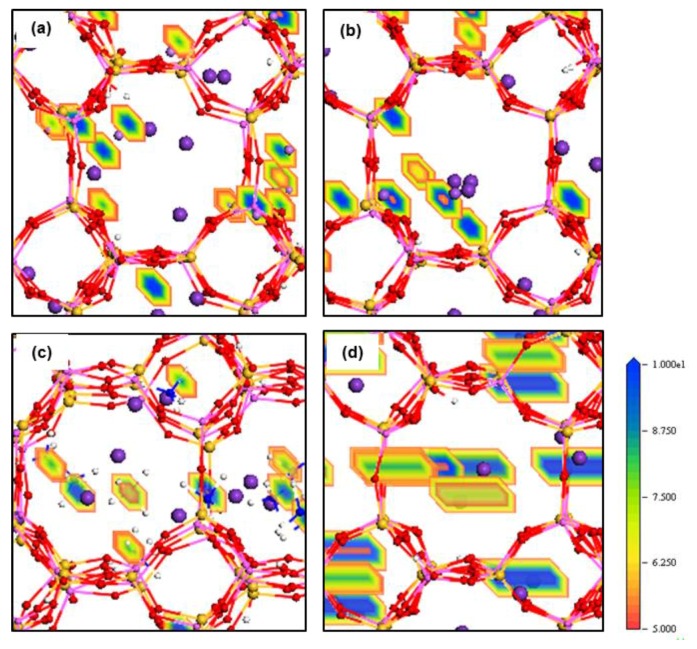
Density field maps of M^n+^ guest cations in (**a**) K^+^/Li^+^, (**b**) K^+^/Na^+^, (**c**) K^+^/NH_4_^+^ and (**d**) K^+^/Cs^+^ systems retained inside ZM-110 after 8.5 ns MD simulations.

The density field maps of K^+^ and M^n+^ guest cations inside ZM-001 and ZM-110 are illustrated in Figure 4 and Figure 5, respectively (as well as Figures S4 and S5). The ion density field maps in both ZM-001 and ZM-110 show that K^+^, NH_4_^+^, Rb^+^ and Cs^+^ cations occupy both the middle of channels and close to cage surfaces. However, Li^+^, Na^+^, Mg^2+^ and Ca^2+^ cations prefer close to cage surfaces.

The RDFs of guest cations with O, Si and Al framework atoms, Cl^−^ and oxygen of water molecules inside membranes are calculated and shown in Figures S6 and S7. The first peak of g(r) shows the nearest distance of guest cations to the framework atoms. These nearest distances are listed in Table 2. The peak intensities of g(r) show the strength of the interaction between atom pairs and a number of strong peaks show regular arrangements of atom pairs.

The RDF results in Table 2 show that K^+^ cations are at the same distances to the framework atoms, chloride ions and water molecules inside both membranes. The average distances for O-K^+^, Si-K^+^, Al-K^+^, Cl^−^-K^+^ and Ow-K^+^ are 2.43, 3.13, 3.19, 2.58, and 2.94 Å, respectively. The O-M^n+^ distances of guest cations in ZM-001 and ZM-110 are shorter than Si-M^n+^ and Al-M^n+^ distances. Also, the M^n+^ guest cations are closer to framework oxygen atoms compared to Cl^−^ ions and oxygen of water molecules inside membranes.

In addition to the first peaks, another one or two noticeable strong peaks, with equal or higher g(r) intensities, are observed for Li^+^, Na^+^, Mg^2+^ and Ca^2+^ arrangements around framework Si and Al atoms (Figures S6 and S7). 

### 2.3. Ion mobility

**Figure 6:**
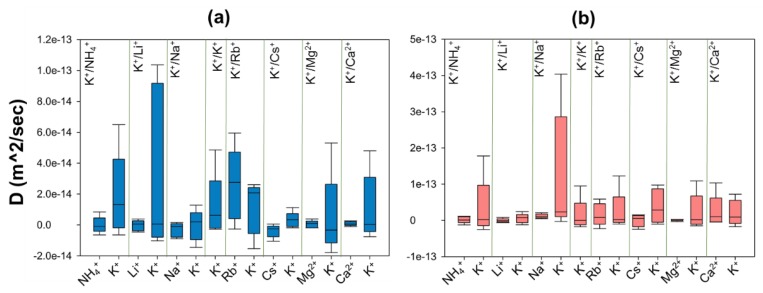
The diversity of self-diffusion coefficients for K^+^ and M^n+^ guest cations of each exchanging system inside (**a**) ZM-001 and (**b**) ZM-110 membranes.

The MSDs of ions inside and outside of membranes computed from five different simulations were used to determine the average MSD for each ion. The self-diffusion coefficient, D, for each ion is estimated from the slope of the average MSD. The D values of ions inside and outside of the membranes are compared and presented in Table 3, Figure 6 and Figure S8.

These results show that ions are more mobile inside ZM-110 compared to ZM-001 except for Rb^+^ which moves faster in ZM-001. Moreover, guest ions are less mobile inside membranes compared to extra-framework K ions, except for the K^+^/Rb^+^ system in ZM-001 and K^+^/Ca^2+^ systems in ZM-110. The self-diffusion of NH_4_^+^, Li^+^, Na^+^ and Cs^+^ cations are negative inside ZM-001 as well as Li^+^ and Cs^+^ D values inside ZM-110. The mobility of ions in ZM-001 are in the order Cs < Na < Li < NH_4_ < Mg < Ca < K < Rb. The relative ion mobility in ZM-110 is in the order Cs < Li < Mg < NH_4_ < Na < K < Rb < Ca. 

The ion D values indicate that all ions are more mobile in the electrolyte outside ZM-001 compared to ZM-110, as a result of the larger simulation box for the ZM-001 system. The K mobility in the electrolyte is higher than guest cations of each system outside ZM-001 and ZM-110, except for the K^+^/Rb^+^ system outside ZM-110. 

## 3. Discussion

**Figure 7:**
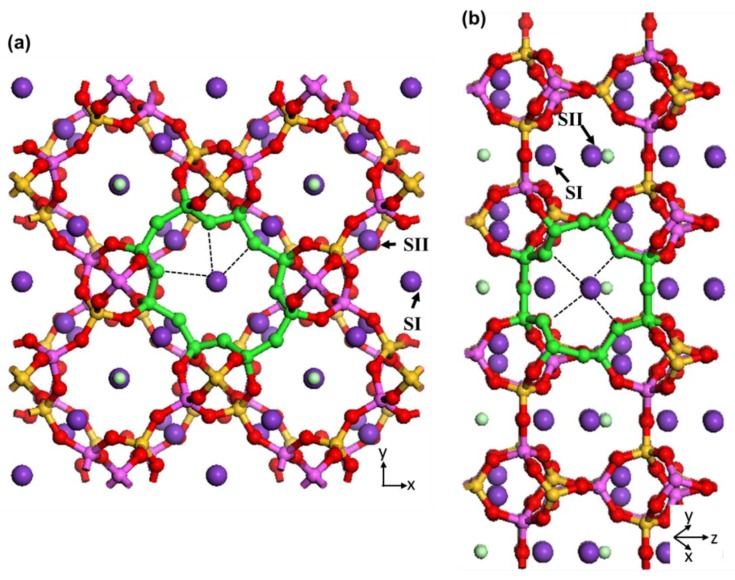
Illustrations of SI and SII sites for extra-framework K in zeolite N supercells along (**a**) [001] and (**b**) [110] crystallographic directions. The 8-membered ring pore openings in each channel direction are highlighted with green colour. The black dashed lines indicate the interaction of potassium cations in sites SI and SII with framework oxygen atoms. Atoms are coloured as silicon = yellow, aluminium = pink, oxygen = red, potassium = purple and chloride = light green.

Zeolite N is built from chains consisting of one-dimensional Periodic Building Units (PBU). These tetrahedral PBUs consist of 5T units (T can be Si or Al) connected together by bridging oxygen atoms along the *a* and *b* axes, translated along the *c* axis to make connected channels. Zeolite N has channels of 3.6 A along the [001] direction interconnected with channels along the [110] direction with different eight-membered ring pore opening shapes and sizes (2.5Å) (Figure 7). 

The intersection of these two channels creates cages that surround the potassium and chloride extra-framework ions and water molecules. Two different T sites for Si and Al framework atoms (T1 and T2), and five for the O atoms constructing the framework, are identified by X-ray diffraction [2]. Two different sites are identified for the potassium extra-framework cations (SI and SII) [2]. SI is located in the middle of the eight-membered rings along the [001] direction (K1) and SII is located in the middle of the other eight-membered rings along the [110] direction. Figure 7 illustrates the position of these sites in the zeolite N unit cell. The extra-framework K1 has interaction with three framework oxygens (noted by black dashed lines in Figure 7a), one chloride and two oxygens of water molecules. The extra-framework K2 has interaction with four framework oxygens (noted by black dashed lines in Figure 7b), one chloride and two oxygens of water molecules. These loose van der Waals interactions can easily breakdown during exchange processes resulting in potassium cations leaving their structural sites for locations of more favourable energy for zeolite N. 

As mentioned in the Introduction, experimental investigations present valuable data on the ion-exchange capability and comportment of zeolite N. However, the exchange mechanism of cations within the zeolite N structure is unclear. In this study, we investigated the ion exchange characteristics of mono- and divalent cations in the zeolite N structure by molecular dynamics calculations. Here, we discuss the retention, structural arrangement and mobility of monovalent NH_4_^+^, Li^+^, Na^+^, K^+^, Rb^+^ and Cs^+^ as well as divalent Mg^2+^ and Ca^2+^ cations inside zeolite N membranes. This allows exploration of the exchange and diffusion mechanism of cations inside channels along the [001] and [110] directions of zeolite N.

### 3.1. Ion Retention

The chemical formula of the zeolite N unit cell indicates 12 exchangeable cations to compensate for the negatively charged −10e of the aluminosilicate framework and two Cl anions [2]. The results show that the total charge compensation on cations per unit cell in most exchange systems is below 12, except for Li^+^ in ZM-001 and Na^+^ and Li^+^ in ZM-110. The potassium-rich ZM-001 and ZM-110 membranes without guest cations, release 8% and 11%, respectively, of their K to solution and thus, contain less than 12 cations per unit cell. Over the simulation time, each membrane loses up to one K^+^ per unit cell. This calculated outcome is consistent with experimental observations that show a neutral water solution will record a pH ~ 9 (or greater) with addition of zeolite N at room temperature. Measurements of this solution with zeolite N also show the presence of K^+^ ions. 

Our previous simulation outcomes [10] show similar conditions regarding the total number of cations retained in the membrane. These results suggest that the zeolite N structure prefers to hold no more K or other cations than an approximately equilibrium value. The one exception to this preference is for the small cation, Li^+^, for which zeolite N is able to adsorb more ions than the charge compensating capacity. 

Experimental studies provide valuable data on the tendency of zeolite N to uptake ammonium ions from aqueous solutions [5,6,9]. Moreover, Zwingmann et al. [7] showed that ammonium exchanged zeolite N is an ideal slow release fertiliser for sandy soils due to the high retention capacity of zeolite N for NH_4_^+^ ions. Our computational results from previous [10] and this study for NH_4_^+^ retention are consistent with these experimental results. This study shows that NH_4_^+^ has the highest retention between all exchanging systems in zeolite N membranes along both crystallographic directions. The NH_4_^+^ retention in ZM-110 is higher than ZM-001 (Figure S1a,b). However, the total number of cations that remain in the structure are similar for both membranes. The small sized channel openings in ZM-110 does not allow NH_4_^+^ ions to leave the membrane and thus, ensures capture in 3D cages formed at the intersection of the (001) and (110) planes. Release of K^+^ ions from the membrane provides additional space for retained NH_4_^+^ ions. Our calculations suggest that only NH_4_^+^ ions close to the membrane surfaces can leave the ZM-110 structure. In contrast, NH_4_^+^ ions can more readily leave the ZM-001 membrane due to the larger size of the channel openings.

Li^+^ and Na^+^ are the next monovalent ions that show high and similar retention behaviour in membranes. The K^+^/Li^+^ and K^+^/Na^+^ systems in this study hold more cations compared to other systems due to the small size of Li^+^ and Na^+^ ions. In the ZM-001 membrane Li^+^ and Na^+^ show similar retention rates (Figure S1a,b). The retention of Na^+^ in the ZM-001 membrane is the same as simulations shown previously under similar conditions [10]. However, the K^+^/Li^+^ system retains more K^+^ in the membrane compared to the K^+^/Na^+^ system during the simulation. In the ZM-110 membrane, Na^+^ retention is slightly higher than Li^+^. However, both K^+^/Li^+^ and K^+^/Na^+^ simulations show that a similar amount of K^+^ is retained in the ZM-110 membrane. 

The K^+^/Rb^+^ and K^+^/Cs^+^ systems show the lowest retention and total number of cations per unit cell among monovalent cations in both membranes due to the large size of these ions. The retention of Rb^+^ and Cs^+^ is higher in ZM-110 than ZM-001 (Figure S1a,b). Similar to the case for NH_4_^+^, the large size of these ions does not allow passage through the small opening of the ZM-110 membrane channels to the solute. The retention behaviour of Rb^+^ and Cs^+^ compared to other monovalent cations in this study for ZM-001 is similar to our previous outcomes [10]. However, in this study, we found lower retention ratios over time for Rb^+^ and Cs^+^.

Experimental data show that the potassic form of zeolite N can take up to three times more Ca^2+^ than Mg^2+^ from a mixed solution of NH_4_^+^, Mg^2+^ and Ca^2+^ (with low ammonium concentration, 30 mg/L) and this uptake of divalent ions is 10× lower than the NH_4_^+^ uptake [9]. These data indicate a high preference of zeolite N for monovalent cations over divalent ions. Computational results from this study for retention of Mg^2+^ and Ca^2+^ ions in zeolite N are in good agreement with experimental outcomes [9]. For example, the number of guest cations for the K^+^/Mg^2+^ and K^+^/Ca^2+^ systems show the lowest value for total retained cations in the membranes (Figure S1a,b). Accordingly, in these systems the number of K^+^ ions retained in the membrane are higher than Mg^2+^ or Ca^2+^. 

Based on the experimentally determined chemical formula for zeolite N, each unit cell contains two Cl^−^ anions [2]. However, our simulations show that both membranes release up to one Cl^−^ per unit cell into solution during the exchange process (Figure S1e,f). A larger cation size results in greater reduction in the number of chlorides inside each membrane. Consistent with this, systems with a lower amount of cations per unit cell require a lower (or equivalent) amount of anions. These results are in also agreement with experimental data [9]. 

Experimental and computational studies show that hydrated zeolite N contains 8 water molecules per cage [2,10]. The results from this study reveal that zeolite N membranes along different directions show different hydration behaviour during the exchange process. For example, the number of water molecules per cage changes during the ion-exchange process depending on the guest ions. A ZM-001 membrane releases zeolitic water into solution at early stages of the exchange process in all K^+^/M^n+^ systems and subsequently continuously adsorbs water molecules over time. This feature is noteworthy for the K^+^/Cs^+^ system of the ZM-001 membrane. Concentration profiles confirm that this increase is due to adsorbed water molecules in the opening of the pores to the membrane. However, the total number of water molecules inside the membrane cages is constant (Figure S9). All exchanging systems in ZM-110 adsorb more than 8 water molecules per cage during the simulation except for the K^+^/Rb^+^ and K^+^/Cs^+^ systems. Zeolite N channels are not large enough to accommodate K^+^, Rb^+^ and Cs^+^ cations together with water molecules. As with ZM-001, water molecules concentrate at the pore openings of the zeolite N membrane. 

### 3.2. Ion Localization

Ion concentration profiles, electron density field maps and RDF results provide different perspectives on the localization of guest and host ions within zeolite N membranes.

The concentration profiles show that ions undergo similar localisation behaviour within the ZM-001 and ZM-110 membranes (Figure 2 and Figure 3, Figures S2 and S3). The ionic size predominantly affects the distribution of ions inside membranes whereby larger ions, such as NH_4_^+^, K^+^, Rb^+^ and Cs^+^, are distributed more uniformly compared with smaller ions such as, Li^+^, Na^+^, Mg^2+^ and Ca^2+^. On the other hand, these simulations show that NH_4_^+^, Li^+^, Na^+^, Mg^2+^ and Ca^2+^ ions concentrate in channel openings at the surface of the membrane due to their strong interaction with framework oxygens. 

The electron density field maps indicate that for ion localisations inside both types of membrane cages, K^+^ and NH_4_^+^ ions localise at SI, SII and slightly shifted positions close to these two sites (Figure 4 and Figure 5, Figures S4 and S5). However, ions larger than K^+^, Rb^+^ and Cs^+^, exactly occupy both SI and SII sites. Ions smaller than K^+^, including Li^+^, Na^+^, Mg^2+^ and Ca^2+^, reside in disordered crystallographic positions closer to the framework rather than at crystallographic sites. These ions localise differently in ZM-001 and ZM-110. The Li^+^, Na^+^, Mg^2+^ and Ca^2+^ ions occupy disordered crystallographic positions in cages of ZM-001 that are closer to SII sites. However, their locations in ZM-110 cages are closer to SI sites. The Na^+^ and Ca^2+^ ions are located at further distances to the framework compared to Li^+^ and Mg^2+^ ions, due to their comparatively larger ionic size.

The RDF values in Table 2 indicate that all guest cations have stronger interaction with framework oxygen than oxygen of the water molecules or with Cl^−^ anions inside the membranes. The RDF results show that K^+^ ions are located at the same distances to the framework in all exchanging systems, though the membranes contain different guest ions with different ionic sizes. The average calculated K distances to O, Si and Al of the framework, Cl anions and oxygen of water molecules inside the membranes are in good agreement with XRD data [2].

The nearest distance of M^n+^ cations to the framework atoms are identified by the position of the first peak of the function g(r). The distances of M^n+^ cations to the zeolite N framework atoms of ZM-001 and ZM-110 membranes are similar except for Rb^+^ and Cs^+^ cations. In ZM-110, most Rb^+^ and Cs^+^ cations tend to locate in the middle of cages at a further distance to the framework. However, in ZM-001 these ions are equally localised at both sites. 

The nearest distances for NH_4_^+^ to the framework oxygen and to Si or Al atoms of ZM-001 are larger and smaller, respectively, than previously obtained values [10]. However, the RDF distances for Na^+^, K^+^, Rb^+^ and Cs^+^ are smaller than our previous results [10]. These differences in nearest distance of atom pairs between these models of zeolite N exchange, are related to different partial charges on framework atoms used in simulations [12].

Furthermore, the RDF graphs in this study show notable first peaks for Li^+^ as well as for Na^+^, Mg^2+^ and Ca^2+^ around the framework Si and Al atoms (Figures S6 and S7). These nearest distances for Li^+^, Na^+^, Mg^2+^ and Ca^2+^ to framework Si and Al atoms are due to the small sizes of these cations as well as to the presence of two different Si and Al atomic positions (T1 and T2 sites) with different partial charges in the zeolite framework [12]. RDF plots and density field maps indicate that these ions are closer to the Si/Al in T2 sites rather than the Si/Al in T1 sites.

The strength of the interaction between atom pairs is estimated from peak intensities of g(r). The peak heights for O-M^n+^ for NH_4_^+^, Li^+^, Na^+^, Mg^2+^ and Ca^2+^ are higher than Si/Al-M^n^. Moreover, the O-M^n+^ value for these ions is the nearest distance to the framework atoms that show strong interaction with framework oxygen atoms compared to Si and Al. The interaction increases from NH_4_^+^ < Na^+^ < Ca^2+^ < Li^+^ < Mg^2+^. In contrast, the higher peaks for Si/Al-M^n+^ where M = K^+^, Rb^+^ or Cs^+^ are not the nearest distances and consequently have weaker interaction with framework oxygen atoms. The interaction strength decreases from K^+^ > Rb^+^ > Cs^+^, in complete agreement with previous outcomes [10]. RDF results using ion density profiles and ion density fields confirm that ions are localised inside the framework relative to their ionic size. An exception to this outcome is NH_4_^+^ for which hydrogen bonding provides stronger interactions with framework oxygen atoms compared with other cations evaluated in these simulations [10]. 

### 3.3. Ion Diffusion

We investigate the relative mobility of ions inside and outside zeolite N membranes by calculating the self-diffusion coefficient (D) of ions from their mean square displacement (MSD) over the simulation time. The diffusion behaviour of ions inside the confined geometry of zeolites is clearly very different from their bulk behaviour in solution. Simulations show that values of D for ions inside zeolite N membranes are smaller than values obtained in the electrolyte solution by several orders of magnitude (Figure 6 and Figure S8). 

The D values for cations inside both membranes are close to zero. Moreover, the measured D values for NH_4_^+^, Li^+^, Na^+^ and Cs^+^ cations are negative inside ZM-001 as well as for Li^+^ and Cs^+^ inside ZM-110. Close inspection of MSD curves reveal that ions do not show diffuse behaviour while some curves show different behaviour regimes over time with positive and negative slopes. This behaviour means that movement of ions inside the membranes is significantly affected by a number of mechanisms. The small or negative diffusion of ions in this study indicates that these ions can not pass the free energy barriers inside zeolite N channels and jump from one low energy site to another. Therefore, these ions localise in a specific position within the structure and show an oscillatory behaviour. 

These energy barriers are present in all directions and include dispersion-repulsion and electrostatic energies between ions and the framework as well as the activation energy that a particular ion requires to move between different crystallographic positions [13]. The density field illustrations shown in Figure 4 and Figure 5, Figures S4 and S5 are exemplars of this mechanistic interpretation for zeolite N. No systematic dependence on ionic size is observed for D values of ions inside zeolite N membranes. 

There are few experimental studies calculating the self-diffusion of cations inside different zeolites (e.g., analcite [14], chabazite [15], mordenite [16] and clinoptilolite [17]) by measuring all activation, dispersion-repulsion and coloumbic energies. These studies calculated the self-diffusion coefficient values for monovalent cations NH_4_^+^, Na^+^, K^+^, Rb^+^, and Cs^+^, in different zeolites, in the range of 10^−11^−10^−26^ m^2^·sec^−1^ and for divalent cations in the range of 10^−15^−10^−17^ m^2^·sec^−1^. The self-diffusion coefficient values for cations obtained in this study are of similar magnitude to experimentally measured values of self-diffusion for cations in other zeolites at 25 °C [14,15,16,17]. 

### 3.4. Ion Exchange Mechanism

In general, the ion exchange property of zeolites and of cation diffusion in zeolites depend on various parameters, including cation size, incipient charge on cations, the smallest free diameter of channel pore-openings, the number of oxygens involved in the pore-openings, the Si/Al ratio and finally, the water flux inside the zeolite structure and its interaction with cations. Therefore, the difference between ZM-001 and ZM-110 ion exchange properties is due to the difference between their pore-opening diameter and the shape of the pore-opening that interfaces with the ambient solution, since all other parameters are the same for both membrane types. However, the interconnection of [001] and [110] channels in both membranes complicates the intracrystalline exchange and diffusion of cations.

Ammonium ions have the highest retention in these simulations and the closest distances to framework atoms in both membranes, even though the ionic size for NH_4_^+^ is larger than K^+^. In our previous simulations, we indicated that hydrogen bonding influences the selectivity of NH_4_^+^ in comparison to other monovalent cations [10]. The hydrogen in NH_4_^+^ interacts with between one and three framework oxygen atoms and between one and two water molecules or other NH_4_^+^ ions. Of these, the interaction between the NH_4_^+^ hydrogen and framework oxygen atoms is the most stable. 

These hydrogen bonds result in completely different diffusive behaviour for NH_4_^+^ compared to other cations. The formation and elimination of hydrogen bonding facilitates the movement of NH_4_^+^ ions within zeolite channels. However, K^+^ cations leave the membranes more quickly than NH_4_^+^ ions due to loose van der Waals interactions with framework and water molecules. As we have seen, the self-diffusion of K^+^ cations is higher than NH_4_^+^ ions in K^+^/NH_4_^+^ systems. 

In this study, we consider nitrogen as the centre of the NH_4_^+^ ion for calculation of the ammonium self-diffusion. The D value for the total NH_4_^+^ ion is larger than the D value of N by several orders of magnitude, as a result of changes in hydrogen bonding. For example, the nitrogen and total NH_4_^+^ self-diffusions in ZM-001 are −1.1 × 10^−17^ m^2^·sec^−1^ and 7.2 × 10^−12^ m^2^·sec^−1^, respectively. Although the NH_4_^+^ interaction is the same in both membranes, the ZM-110 membrane retains more NH_4_^+^. The small diameter of channels along the [110] direction increases the energy barrier for NH_4_^+^ ions leaving the membrane with water molecules. Therefore, NH_4_^+^ ions localise within ZM-110 membrane channels along the *a* axis with limited or no access to the solution outside the membrane.

In general, the exchange of monovalent cations and their location to the framework inside zeolite N membranes depends on their ionic size and, respectively, decrease and increase with increase in cation size. However, the exchanges of Li^+^ and Cs^+^ do not follow this general principle for the ZM-110 membrane. Li^+^ and Na^+^ cations have lower mobility compared to K extra-framework cations, due to their stronger electrostatic interactions with the zeolite N framework. The Li^+^ cations show higher levels of interaction than Na^+^ cations due to a higher charge density. Na^+^ cations show between two and three electrostatic interactions with framework oxygen atoms, one van der Waals interaction with water molecules and/or one interaction with chloride anions. However, Li^+^ cations show interactions with two water molecules while they have the same number of electrostatic interactions with framework oxygen and chloride atoms. As a result, Li^+^ can be more mobile within zeolite N channels compared to Na^+^. 

Rb^+^ and Cs^+^ ions seem to follow similar diffusion mechanisms in zeolite N. These cations prefer to localise at the SI and SII sites, which are the lowest energy sites within the zeolite N structure. Rb^+^ and Cs^+^ have between three and four van der Waals interactions with framework oxygens and between two and three interactions with water molecules and/or one chloride ion. As a result of these many interactions, and their large ionic size, these ions oscillate at their site positions and show limited diffusivity. In comparison to K^+^, Rb^+^ shows a higher self-diffusion value in the ZM-001 membrane. The diffusion of Rb^+^ and Cs^+^ in the ZM-110 membrane is anisotropic. Rb and Cs in the ZM-110 membrane cannot transport through channels to the external solution due to their ionic size in comparison to the small pore opening sizes of this membrane direction. Thus, these larger ions prefer to move through channels along the *a* axis inside the ZM-110 membrane. This attribute is evident by the rectangular shape of the density fields for these cations shown in Figure 4d and Figure S5b.

The behaviour of divalent Mg^2+^ and Ca^2+^ cations gives the impression that they follow a similar exchange and diffusion mechanism as monovalent Li^+^ and Na^+^ cations, respectively. As shown in Figure 4 and Figure 5, Figures S4 and S5, the localisation behaviour and structural arrangements around framework oxygen atoms are similar, especially for Mg^2+^ and Li^+^. However, the exchange and diffusion processes are completely different. For example, the Mg^2+^ and Ca^2+^ mobilities inside zeolite N membranes are higher than monovalent Li^+^ and Na^+^ cations even though they show the same number of electrostatic interactions with framework oxygen atoms as Li^+^ and Na^+^. However, Mg^2+^ and Ca^2+^ interact with more water molecules compared to Li^+^ and Na^+^. As a result, these associated water molecules enhance the mobility of Mg^2+^ and Ca^2+^ inside zeolite channels. Nevertheless, in comparison to extra-framework K^+^, the diffusion of Mg^2+^ and Ca^2+^, is less facile due to the higher coulombic interaction with framework oxygen atoms. Thus, for the ZM-001 membrane, these simulations suggest that divalent Mg^2+^ and Ca^2+^ show analogous retention behaviour to monovalent Li^+^ and Na^+^.

## 4. Materials and Methods 

The primary unit cell for zeolite N, used in this study, is based on the crystal structure defined by Christensen and Fjellvag [2] using synchrotron X-ray powder diffraction. The Materials Studio (version 18.1), suite of programs (Dassault Systèmes BIOVIA, San Diego, CA, USA) [18] is used to construct the zeolite N models, DFT calculations and subsequent MD simulations.

### 4.1. DFT Calculations

The partial charges of zeolite N framework atoms are calculated by periodic DFT methods [19] on a zeolite N unit cell without extra-framework atoms and water molecules. The geometry optimization and population analysis is obtained using the GGA-PBE functional [20,21] with double numerical plus polarization (DNP) basis sets 4.4 [22]. The convergence tolerance criteria are 1 × 10^−5^ Ha, 0.002 Ha/Å and 0.005 Å for energy, force and displacement convergence, respectively. The SCF convergence criterion is set to an energy tolerance 1 × 10^−6^ Ha. The Mulliken partial charges are obtained from population analysis of DMol^3^ [23,24] code (DMol^3^; Accelrys Inc.: San Diego, CA, USA, 2016.) in Accelrys Materials Studio. The geometry optimized unit cell is cleaved along two different planes, (001) and (110), and then capped with –OH groups on both surfaces with a vacuum slab of 5 Å. In order to obtain the partial charges of O and H atoms on the surfaces, the cells are optimized by the DFT model described above. Table S1 represents the calculated Mulliken partial charges for framework atoms, O and H atoms at surfaces. The partial charges of extra-framework atoms are considered equal to their ionic charge. 

### 4.2. MD Simulations

In order to investigate the exchange capability of zeolite N along two different channel directions, two membranes are built along the [001] and [110] directions. A 2 × 2 × 2 supercell is used to make the membrane along [001] and a 2 × 2 × 1 supercell is used to make the membrane along [110]. Both supercells were cleaved and then caped with –OH on the surfaces with vacuum slabs of 20 Å on either side. Thus, two different membranes of zeolite N as shown in Figure 2a and Figure 3a are generated and labelled as ZM-001 and ZM-110. The size of ZM-001 is 19.8 × 19.8 × 28.2 Å^3^ and ZM-110 is 13.1 × 27.9 × 29.6 Å^3^. These two membranes are different sizes in order to maintain equal numbers of framework and extra-framework atoms for both types of membranes. Table S1 presents the number of framework, extra-framework and water molecules in both membranes. Water molecules with a density of one g/cm^3^ are added to either side of the vacuum slab of membrane models. The SPC model of water is used for all simulations.

All MD simulations are conducted using the Forcite Plus module in Materials Studio software package (Dassault Systèmes BIOVIA, San Diego, CA, USA) [18] with COMPASS force field [25]. Prior to all MD simulations, a geometry optimisation with periodic boundary conditions is conducted on zeolite membranes in water. The minimization is carried out by a quasi-Newton procedure with 500 iterations and the same convergence criteria in DFT models are applied. The electrostatic interactions are calculated by Ewald summation with accuracy 1 × 10^−4^ kcal/mol. However, the direct cut-off with 15.5 Å distance is applied for determining the van der Waals interactions. We do not use the Ewald summation for long range interactions in order to decrease the time for computation. In addition, the 15.5 Å distance cut-off is sufficiently accurate compared with a Ewald summation. Initially, the zeolite framework in both membranes is kept rigid to allow extra-framework atoms and water molecules to displace with respect to each other to reach minimum energy. These optimised models are used as starting configurations for further MD simulations. In these MD simulations, all framework, extra-framework atoms and water molecules are released in order to move freely during the simulation. Our previous work [10] shows that the structure of zeolite N framework is quite stable under this condition and shows no significant change.

Both ZM-001 and ZM-110 membranes contain 80 Si and Al atoms, 96 K^+^ and 12 Cl^−^ ions and 128 water molecules. The charge of the framework in each membrane is −80e. In order to investigate the retention of ions inside membranes, a chemical potential is created between the inside and outside of the membranes. The guest M^n+^ (*n* = 1 or 2) cations are placed randomly inside the membranes and 40 chloride ions are distributed in the solvent on either side of the membranes. The number of guest cations depends on their total charge compensating the −80e of frameworks. Therefore, the number of added guest mono-and divalent cations are 80 and 40, respectively. The guest ions include NH_4_^+^, Li^+^, Na^+^, K^+^, Rb^+^, Cs^+^, Mg^2+^ and Ca^2+^ cations. The models created containing guest cations and extra chlorides, are designated as K^+^/M^n+^ systems in the results and discussion sections (represented in Figure 2a and Figure 3a). 

A 30 ps equilibrium MD simulation is performed in an NVT ensemble at 298 K with NHL thermostat [26,27] and with a time step of 1.0 fs followed by a production MD simulation for 8.5 ns. This method allows evaluation of the dynamic, structural and statistics properties as well as ions and water molecule retention inside zeolite N membranes for the full MD simulation. To estimate ion localisation inside and outside the membranes, radial distribution functions (RDF) of atom pairs and concentration profiles of ions inside and outside the membranes as well as the electron density fields of ions inside membranes are determined from the last 1 ns of the MD simulation. The dynamics of ions in the membrane and the solvent are studied by calculating the self-diffusion coefficient (D). This parameter is computed using the mean square displacement (MSD) of ions over time. In order to increase the accuracy of calculations for D, five different 1 ns MD simulations are conducted after 8.5 ns simulations for each K^+^/M^n+^ system and the average MSD of each ion is used to calculate D. The five MD runs used different starting coordinates and random velocities for each simulation. The framework was fixed in these simulations while ions and water molecules were released to move inside and outside the membranes.

## 5. Conclusions

In this study, we have investigated the ion exchange characteristics of zeolite N membranes at atomic scale using molecular dynamics simulations. These membrane models allow exploration of exchange and diffusion mechanisms for univalent or divalent cations within the three-dimensional porous structure of zeolite N.

We demonstrate that the exchange and diffusion of cations varies depending on the nature of cation, ion size and charge as well as the direction of exchange process within zeolite N channels. The NH_4_^+^ diffusion mechanism is considerably different to the mechanism(s) for other cations due to the influence of hydrogen bonding. The diffusion behaviour of guest cations smaller than the extra-framework K^+^ ion is isotropic and, in general, follows a similar mechanism in [001] and [110] directions. However, the diffusion of cations larger than K^+^ is anisotropic in zeolite N due to different diameter channels along the [001] and [110] directions.

Taking into account the retention behaviour and exchange mechanisms of the cations evaluated in these simulations, we suggest that the cation selectivity series for zeolite N as: NH_4_^+^ > Na^+^ > Li^+^ > K^+^ > Ca^2+^ > Rb^+^ > Cs^+^ > Mg^2+^.

Furthermore, we compare the dynamic behaviour of our zeolite N model in this study with previous work [10] in which we use different partial charges for framework atoms. This comparison indicates that the structural arrangements of ions and water molecules inside zeolite N membranes are influenced by the partial charges of framework atoms. However, the general ion selectivity of zeolite N membranes is not altered by the partial charges of framework atoms.

The methodology applied in this study provides a practical method to investigate the ion exchange mechanism within other zeolite structures or to predict their exchange behaviour for ion exchange applications.

## Figures and Tables

**Table 1:** Number of initial and retained ions in K-ZM without guest cations, ZM-001 and ZM-110 membranes as well as their comparison with a previous study [10]. The potassium retained in Site I and Site II after 8.5 ns simulations are as percentages.

	**ZM-001**						**Murthy et al. [10]**		**ZM-110**					
	**Total K^+^**	**%K1**	**%K2**	**M^n+^**	**Cl^−^**	**H_2_O**	**K^+^**	**M^+^**	**Total K^+^**	**%K1**	**%K2**	**M^n+^**	**Cl^−^**	**H_2_O**
Initial	96.0	32.0	64.0	0.0	16.0	128.0			96.0	32.0	64.0	0.0	16.0	128.0
K-ZM	88.1	%96.8	%89.3	0.0	11.0	118.7			85.5	%85.8	%90.7	0.0	12.0	127.9
	**Total K^+^**	**%K1**	**%K2**	**M^n+^**	**Cl^−^**	**H_2_O**	**K^+^**	**M^+^**	**Total K^+^**	**%K1**	**%K2**	**M^n+^**	**Cl^−^**	**H_2_O**
Initial	96.0	32.0	64.0	80/40	16.0	128.0			96.0	32.0	64.0	80/40	16.0	128.0
K^+^/NH_4_^+^	36.3	%39.7	%36.9	53.0	11.0	123.8	33	55	31.7	%35.3	%31.9	56.6	12.0	116.2
K^+^/Li^+^	45.5	%44.2	%49	51.0	14.0	120.3			45.6	%45.6	%48.4	47.5	14.0	143.9
K^+^/Na^+^	42.1	%46.9	%42.4	50.6	11.4	122.1	39	50	46.9	%50.8	%47.9	51.4	14.0	133.6
K^+^/K^+^	89.3	%37.5	%48.8	46.1	10.0	130.8	87		88.8	%34.5	%49.3	46.2	12.2	132.5
K^+^/Rb^+^	44.0	%37.5	%50	41.1	9.0	127.9	37	48	42.3	%46.4	%42.9	45.4	12.0	133.2
K^+^/Cs^+^	43.8	%50.8	%43	40.1	8.0	122.6	41	42.7	40.9	%46.2	%40.9	47.1	13.0	117.1
K^+^/Mg^2+^	43.1	%46.9	%43.9	25.0	10.6	123.6			54.9	%50.2	%60.7	17.9	13.2	147.4
K^+^/Ca^2+^	41.5	%46.3	%41.7	27.0	10.7	132.2			49.4	%43.8	%55.3	22.0	11.0	137.0

**Table 2:** The nearest distances of M^n+^ ions to framework atoms and oxygen of water molecules (O_w_-M^n+^), within membranes and comparison with previous work [10].

	O-M^n+^ (Å)			Si-M^n+^ ( Å)			Al-M^n+^ ( Å)			O_w_-M^n+^		Cl-M^n+^	
System	ZM-001	ZM-110	Murthy et al. [10]	ZM-001	ZM-110	Murthy et al. [10]	ZM-001	ZM-110	Murthy et al. [10]	ZM-001	ZM-110	ZM-001	ZM-110
K^+^/NH_4_^+^	1.52	1.52	1.43	2.37	2.39	2.39	2.5	2.58	2.39	1.78	1.76	2.07	2.08
K^+^/Li^+^	1.64	1.65		2.21	2.39		2.32	2.42		1.95	1.95	2.26	2.34
K^+^/Na^+^	1.97	1.99	2.07	2.73	2.83	2.83	2.71	2.74	2.79	2.25	2.21	2.63	2.63
K^+^/K^+^	2.41	2.43	2.49	3.16	3.16	3.39	3.16	3.17	3.25	2.59	2.57	2.96	2.94
K^+^/Rb^+^	2.62	2.69	2.65	3.22	3.45	3.45	3.25	3.43	3.47	2.8	2.74	3	3.02
K^+^/Cs^+^	2.84	2.89	3.03	3.35	3.6	3.59	3.41	3.67	3.59	2.96	2.92	3.14	3.17
K^+^/Mg^2+^	1.66	1.65		2.36	2.35		2.41	2.51		1.97	1.96	2.32	2.34
K^+^/Ca^2+^	2.02	2.04		2.69	2.64		2.79	2.83		2.29	2.29	2.72	2.66

**Table 3:** Self-diffusion coefficient of ions in ZM-001 and ZM-110 membranes and in the electrolyte solution.

	ZM-001				ZM-110			
	Inside		Outside		Inside		Outside	
System	M^n+^	K^+^	M^n+^	K^+^	M^n+^	K^+^	M^n+^	K^+^
K^+^/NH_4_^+^	−1.1 × 10^−17^	1.9 × 10^−14^	5.76 × 10^−9^	7.85 × 10^−9^	1.55 × 10^−15^	3.35 × 10^−14^	4.96 × 10^−9^	6.18 × 10^−9^
K^+^/Li^+^	−3.1 × 10^−16^	3.37 × 10^−14^	4.81 × 10^−9^	9.46 × 10^−9^	−2.3 × 10^−16^	5.39 × 10^−15^	3.57 × 10^−9^	7.78 × 10^−9^
K^+^/Na^+^	−3 × 10^−15^	−2.1 × 10^−16^	6.66 × 10^−9^	8.78 × 10^−9^	1.2 × 10^−14^	1.23 × 10^−13^	5.51 × 10^−9^	7.37 × 10^−9^
K^+^/K^+^	1.18 × 10^−14^	1.18 × 10^−14^	7.11 × 10^−9^	7.11 × 10^−9^	1.41 × 10^−14^	1.41 × 10^−14^	5.95 × 10^−9^	5.95 × 10^−9^
K^+^/Rb^+^	2.61 × 10^−14^	1.16 × 10^−14^	6.37 × 10^−9^	7.16 × 10^−9^	1.57 × 10^−14^	2.4 × 10^−14^	4.67 × 10^−9^	4.37 × 10^−9^
K^+^/Cs^+^	−3.9 × 10^−15^	3.18 × 10^−15^	3.62 × 10^−9^	5.2 × 10^−9^	−5.6 × 10^−16^	3.84 × 10^−14^	3.05 × 10^−9^	3.78 × 10^−9^
K^+^/Mg^2+^	4.82 × 10^−16^	5.22 × 10^−15^	3.41 × 10^−9^	7.47 × 10^−9^	9.01 × 10^−16^	2.28 × 10^−14^	1.78 × 10^−9^	7.14 × 10^−9^
K^+^/Ca^2+^	8.46 × 10^−16^	1.07 × 10^−14^	3.24 × 10^−9^	6.28 × 10^−9^	2.49 × 10^−14^	2.06 × 10^−14^	1.85 × 10^−9^	4.1 × 10^−9^

## Supplementary Materials

The following are available online, Table S1: The Mulliken partial cherges, force field assigned types used in this study; Figure S1: The retention of guest cations, chlorides and water molecules in ZM-001 and ZM-110 membranes; Figures S2 and S3: ion concentration profiles along *z* direction of ZM-001 and ZM-110 membranes, respectively; Figures S4 and S5: Density field maps of cations retained inside ZM-001 and ZM-110 membranes, respectively; Figures S6 and S7: RDFs, g(r) for guest cations to framework atoms, chlorides and water molecules inside ZM-001 and ZM-110 membranes, respectively; Figure S8: Self-diffusion coefficient of ions (D) inside electrolyte vs. ionic radius; Figure S9: The concentration profile of water molecules along z direction in different time of MD simulations for K^+^/Cs^+^ system of ZM-001.

## References

[1] Barrer R., Hinds L., White E. (1953). The hydrothermal chemistry of silicates. Part III. Reactions of analcite and leucite. J. Chem. Soc..

[2] Christensen A.N., Fjellvag H. (1997). Crystal structure determination of zeolite N from synchrotron X-ray powder diffraction data. Acta Chem. Scand..

[3] Christensen A.N., Fjellvag H. (1999). nuetron powder diferaction study of the dehydration of zeolite N. Acta Chem. Scand..

[4] Mackinnon I.D.R., Barr K., Miller E., Hunter S., Pinel T. (2003). Nutrient Removal from waste water using high performance materials. Water Sci. Technol..

[5] Thornton A., Pearce P., Parsons S.A. (2007). Ammonium removal from digested sludge liquors using ion exchange. Water Res..

[6] Thornton A., Pearce P., Parsons S.A. (2007). Ammonium removal from solution using ion exchange on to MesoLite, an equilibrium study. J. Hazard. Mater..

[7] Zwingmann N., Singh B., Mackinnon I.D.R., Gilkes R.J. (2009). Zeolite from alkali modified kaolin increases NH4+ retention by sandy soil: Column experiments. Appl. Clay Sci..

[8] Zwingmann N., Mackinnon I.D., Gilkes R.J. (2011). Use of a zeolite synthesised from alkali treated kaolin as a K fertiliser: Glasshouse experiments on leaching and uptake of K by wheat plants in sandy soil. Appl. Clay Sci..

[9] Mackinnon I., Millar G., Stolz W. (2006). Aluminosilicated of zeolite N structure. U.S. Patent.

[10] Murthy V., Khosravi M., Mackinnon I.D.R. (2018). Molecular Modeling of Univalent Cation Exchange in Zeolite N. J. Phys. Chem. C.

[11] Ekhteiari Salmas R., Demir B., Yıldırım E., Sirkecioğlu A., Yurtsever M., Ahunbay M.G. (2013). Silver–Sodium Ion Exchange Dynamics in LTA Zeolite Membranes. J. Phys. Chem. C.

[12] Khosravi M., Murthy V., Mackinnon I.D.R. (2020). Evaluation of DFT methods to calculate structure and partial atomic charges for zeolite N. Comput. Mater. Sci..

[13] Smit B., Maesen T.L.M. (2008). Molecular simulations of zeolites: Adsorption, diffusion, and shape selectivity. Chem. Rev..

[14] Barrer R.M., Rees L.V.C. (1960). Self-diffusion of alkali metal ions in analcite. J. Trans. Faraday Soc..

[15] Barrer R., Bartholomew R., Rees L. (1963). Ion exchange in porous crystals part I. Self-and exchange-diffusion of ions in chabazites. J. Phys. Chem. Solids.

[16] Rees L., Rao A. (1966). Self-diffusion of various cations in natural mordenite. J. Trans. Faraday Soc..

[17] Dyer A., White K.J. (1999). Cation diffusion in the natural zeolite clinoptilolite. J. Thermochim. Acta.

[18] Dassault Systèmes (2014). Materials Studio 18.1.

[19] Tsuneda T. (2014). Density Functional Theory in Quantum Chemistry.

[20] Perdew J.P. (1991). Generalized gradient approximations for exchange and correlation: A look backward and forward. Phys. B.

[21] Perdew J.P., Burke K., Ernzerhof M. (1996). Generalized gradient approximation made simple. Phys. Rev. Lett..

[22] Delley B. (2006). Ground-State Enthalpies:  Evaluation of Electronic Structure Approaches with Emphasis on the Density Functional Method. J. Phys. Chem. A.

[23] Delley B. (1990). An all-electron numerical method for solving the local density functional for polyatomic molecules. J. Chem. Phys..

[24] Delley B. (2000). From molecules to solids with the DMol3 approach. J. Chem. Phys..

[25] Sun H. (1998). COMPASS: An ab initio force-field optimized for condensed-phase applications overview with details on alkane and benzene compounds. J. Phys. Chem. B.

[26] Samoletov A.A., Dettmann C.P., Chaplain M.A. (2007). Thermostats for “slow” configurational modes. J. Stat. Phys..

[27] Leimkuhler B., Noorizadeh E., Penrose O. (2011). Comparing the efficiencies of stochastic isothermal molecular dynamics methods. J. Stat. Phys..

