# Supplementary material for: The Exchange Mechanism of Alkaline and Alkaline-Earth Ions in Zeolite N
Source: Molecules. 2019 Oct 10;24(20):3652. doi: 10.3390/molecules24203652 (PMC6832695; doi:10.3390/molecules24203652)
Supplement: Supplementary file 1 [file molecules-24-03652-s001.pdf]

SUPPLEMENTARY MATERIALS

Exchange Mechanism of Alkaline and Alkalineearth Elements in Zeolite N

Monireh Khosravi [1,*], Vinuthaa Murthy [2] and Ian D R Mackinnon [1]

1 Institute for Future Environments and Science and Engineering Faculty, Queensland University of Technology, Brisbane, QLD 4001, Australia
2 College of Engineering, IT and Environment, Charles Darwin University, Darwin, NT 0909, Australia.
* Correspondence: monireh.khosravinasab@qut.edu.au;

Table S1. The Mulliken partial cherges, force field assigned types and number of framework, extra-framework and water atoms used in this study.

Atom	Atomic charges	Force fields assigned	Number of atoms	
			(001)	(110)
Si 1	1.756	si4z	16	16
Si 2	1.718	si4z	64	64
Al 1	1.654	al4z	16	16
Al 2	1.623	al4z	64	64
O	-1.0886	o2z	304	312
O_{-OH}	-0.8453	o2z	32	16
H_{-OH}	0.299	h1o	32	16
K1	+1	k+	32	32
K2	+1	k+	64	64
Cl	-1	cl+	16	16
O_W	-0.82	o2*	128	128
H_W	0.41	h1o	256	256

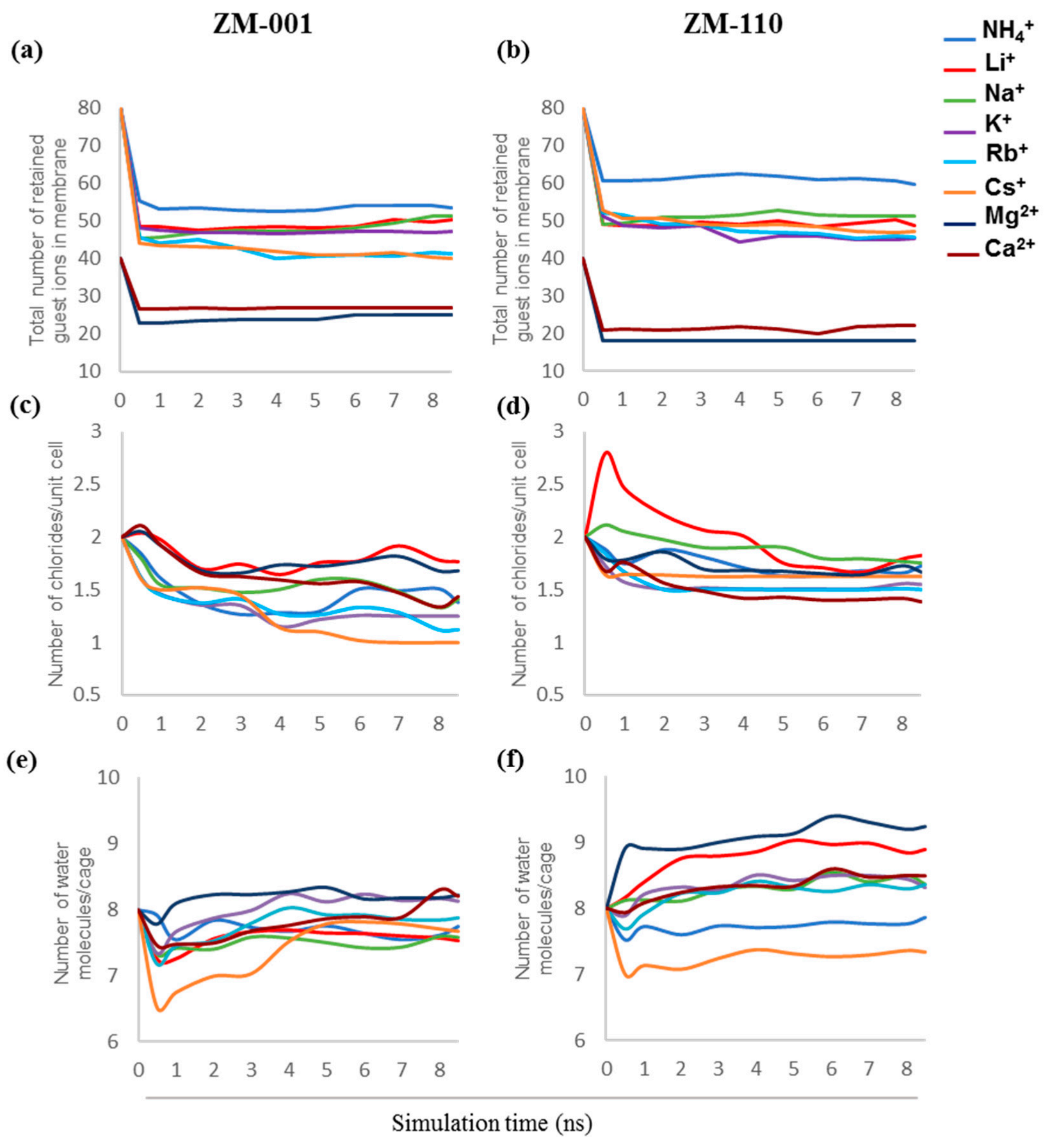

Figure S1. The retention of guest cations in (a) ZM-001 and (b) ZM-110, the number of retained chlorides and per unit cell of (c) ZM-001 and (e) ZM-110; ZM-001 and number of remained water molecules in each cage of (d) ZM-001 and (f) ZM-110.

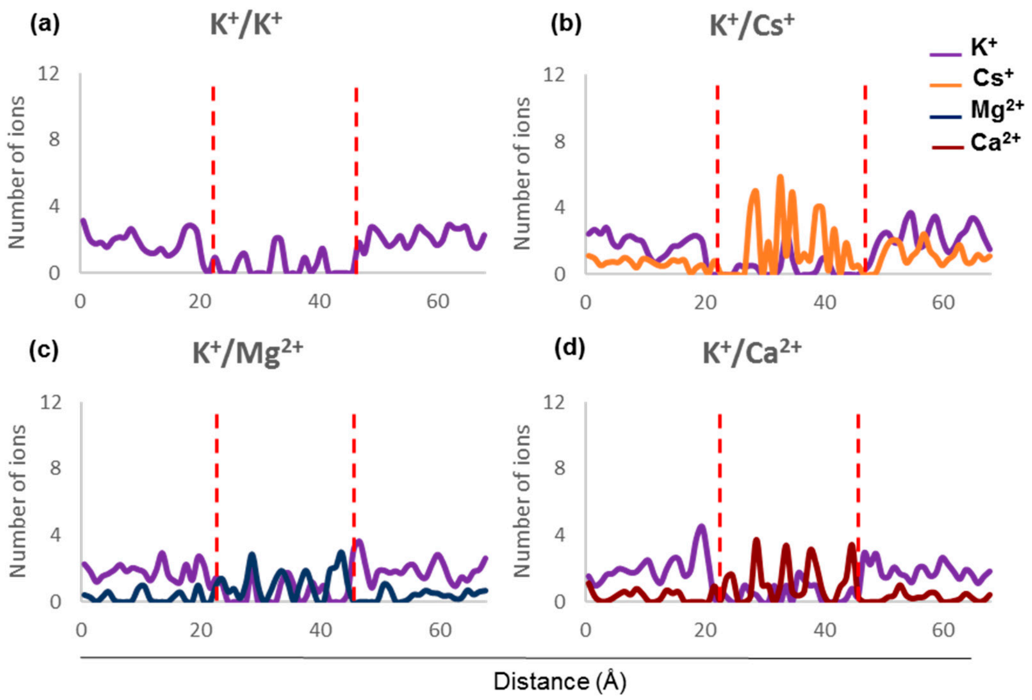

Figure S2. (a-d) ion concentration profiles along *z* direction after 8.5 ns MD simulations. The two red dashed lines indicate the location of ZM-001 surfaces in electrolyte solution.

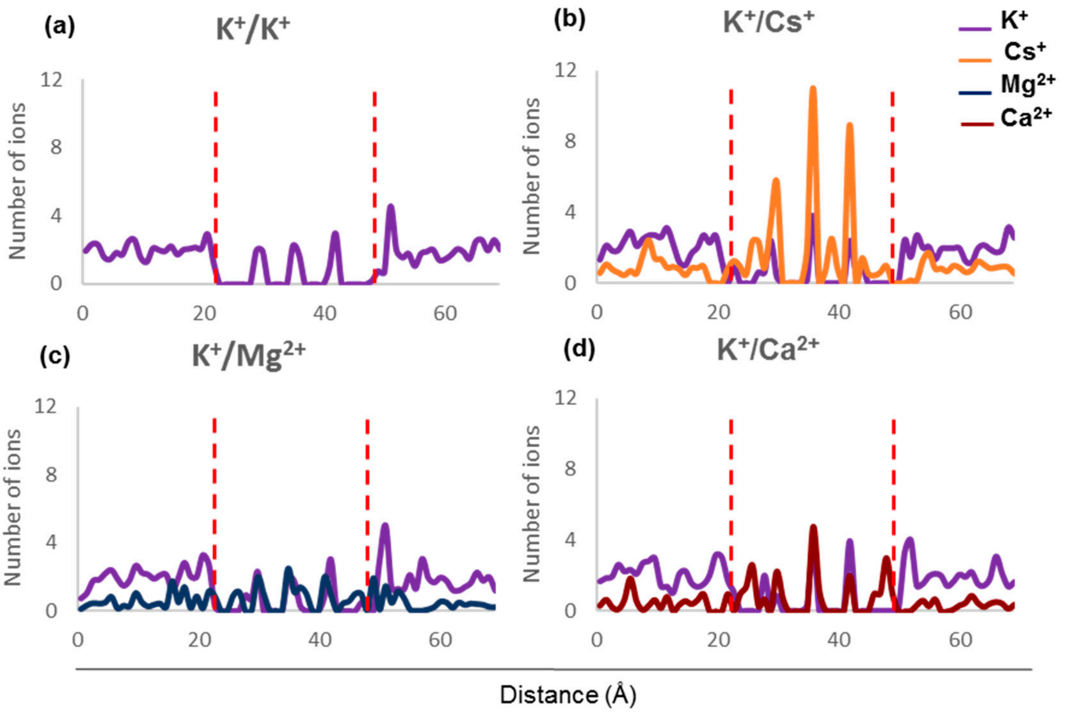

Figure S3. (a-d) ion concentration profiles along *z* direction after 8.5 ns MD simulations. The two red dashed lines indicate the location of ZM-110 surfaces in electrolyte solution.

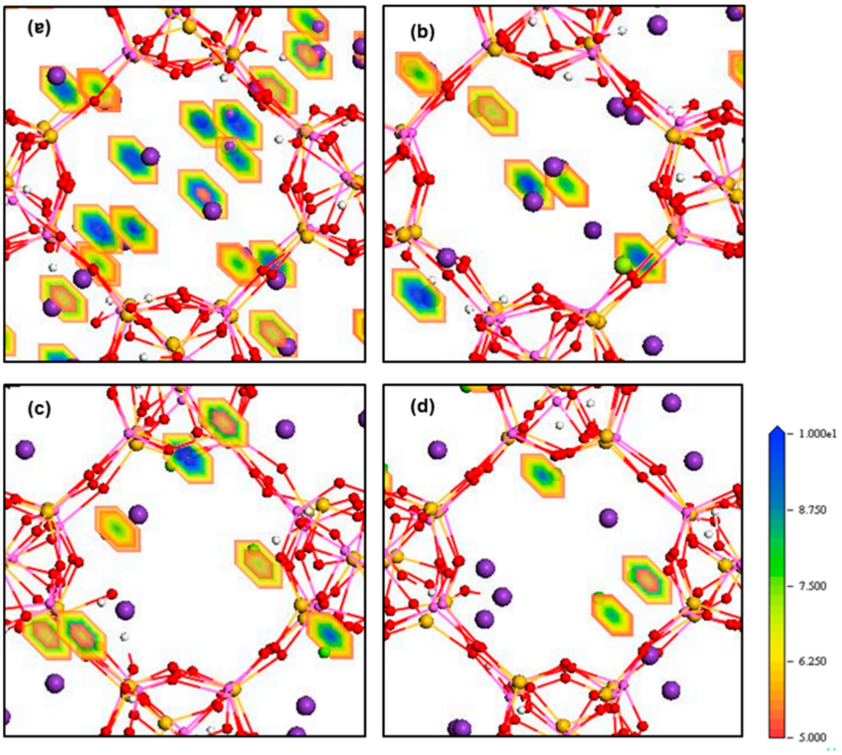

Figure S4. Density field maps of (a) K^+ in K^+/ K^+ system and M^n guest cations in (b) K^+/Rb^+, (c) K^+/Mg^{2+} and (d) K^+/Ca^{2+} systems retained inside ZM-001 after 8.5 ns MD simulations.

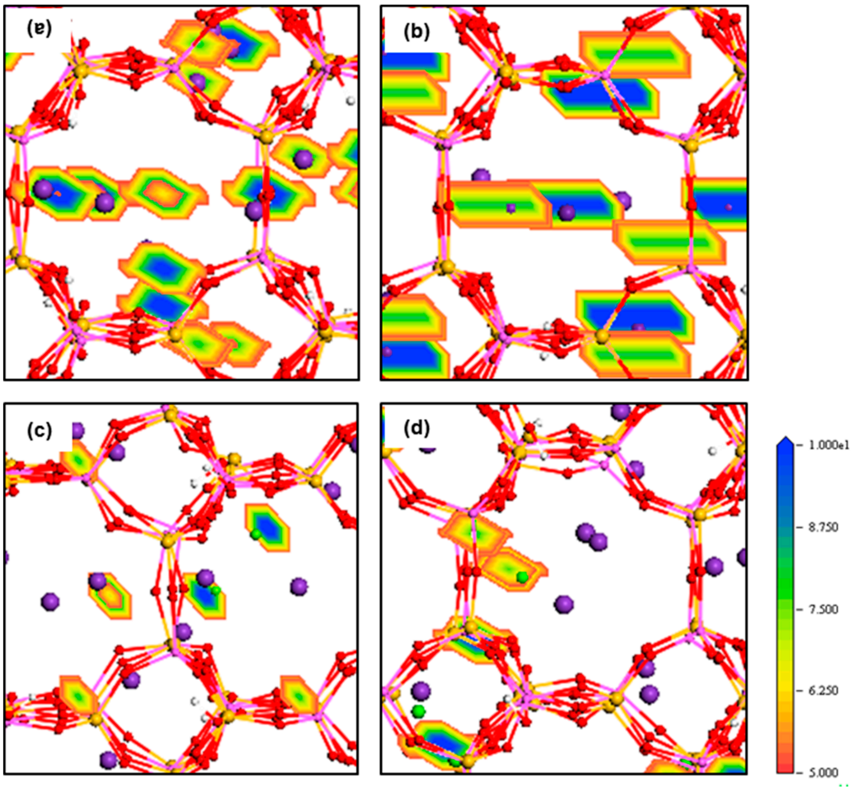

1)

Figure S5. Density field maps of (a) K^+ in K^+/ K^+ system and M^n guest cations in (b) K^+/Rb^+, (c) K^+/Mg^{2+} and (d) K^+/Ca^{2+} systems retained inside ZM-110 after 8.5 ns MD simulations.

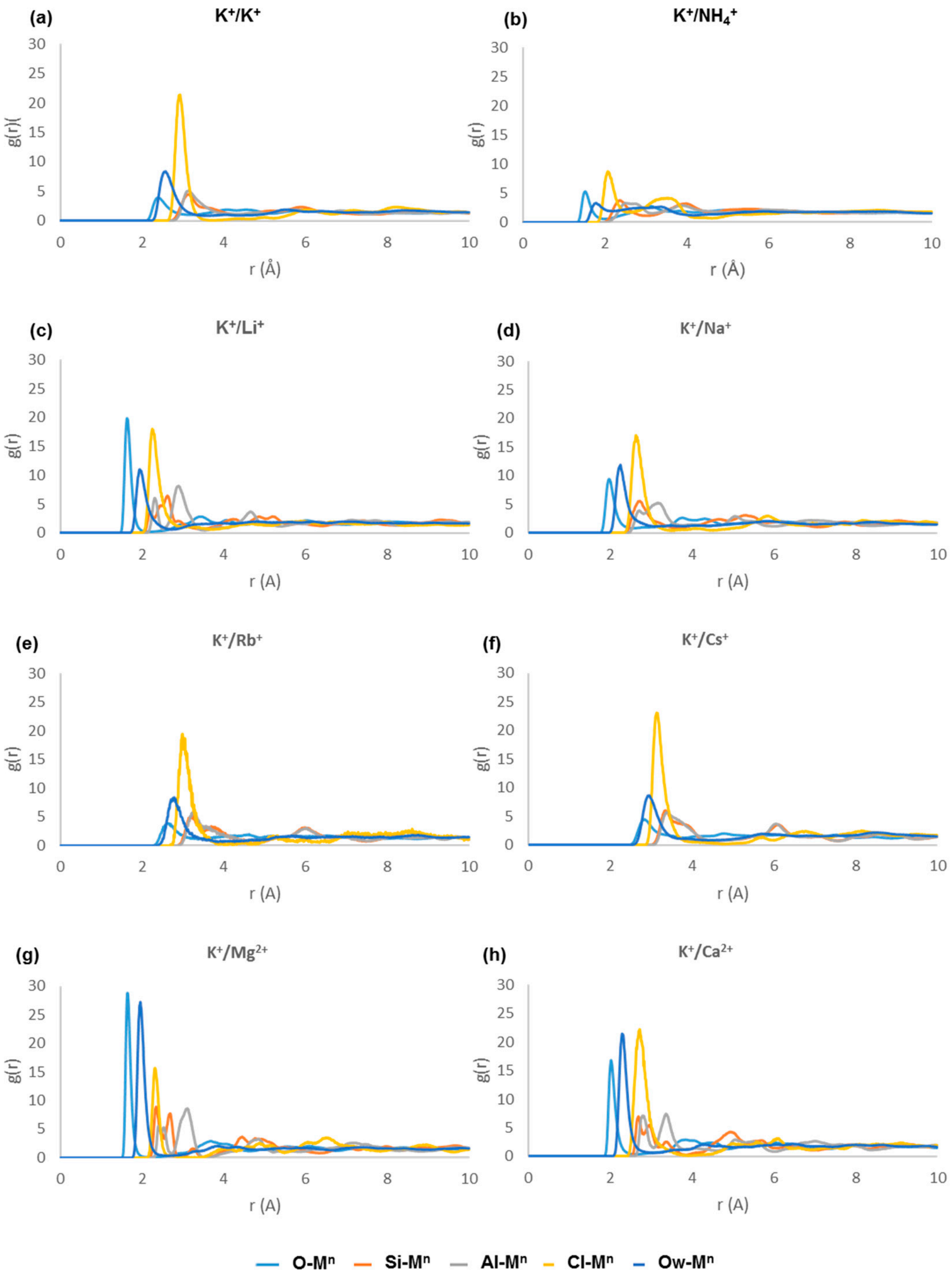

Figure S6. RDFs, g(r) for guest cations to framework atoms, chlorides and water molecules inside ZM-001 membrane.

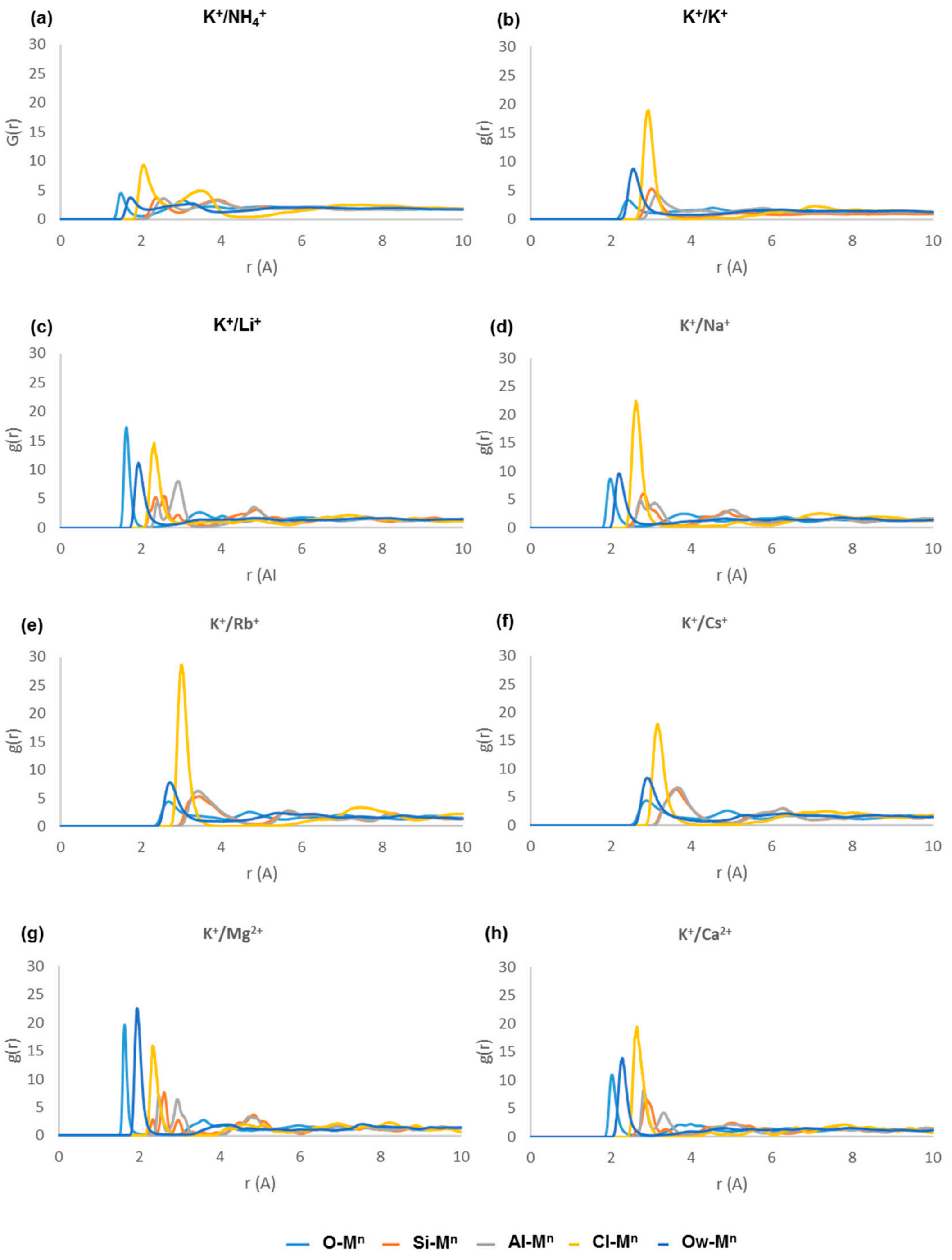

Figure S7. RDFs, g(r) for guest cations to framework atoms, chlorides and water molecules inside ZM-110 membrane.

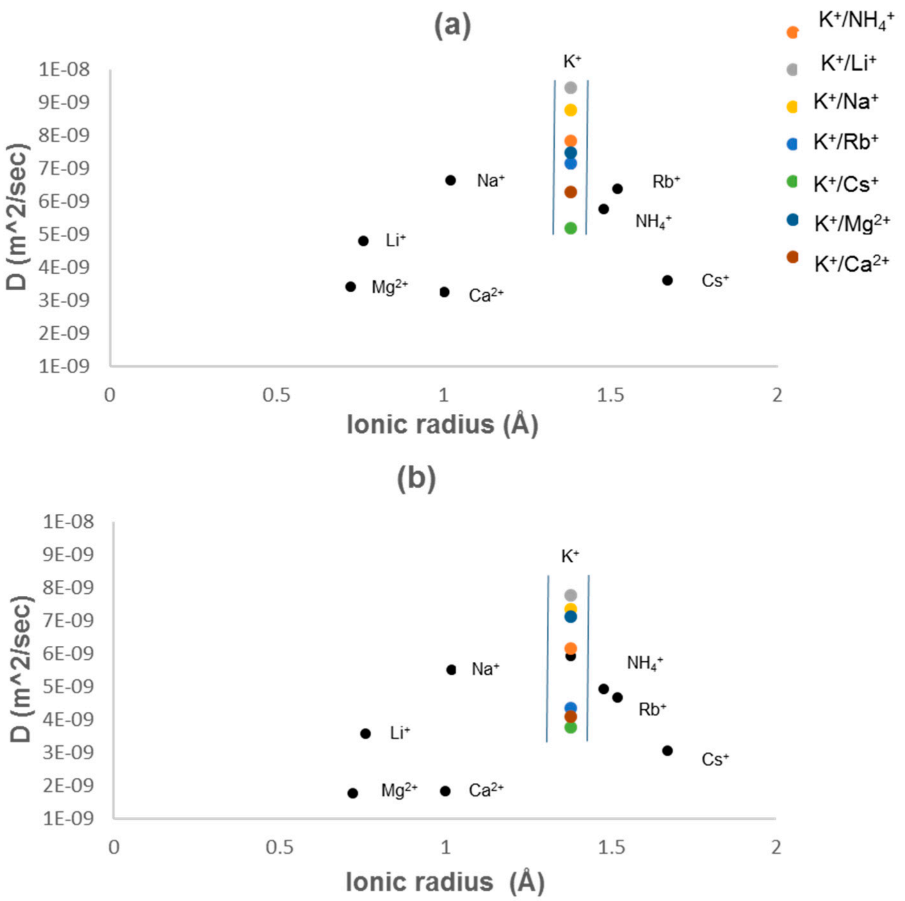

Figure S8. Self-diffusion coefficient of ions (D) inside electrolyte vs. ionic radius. The black labelled points are D values of guest cation in each system. The D values of K cations in electrolyte of each system are identified with different colours.

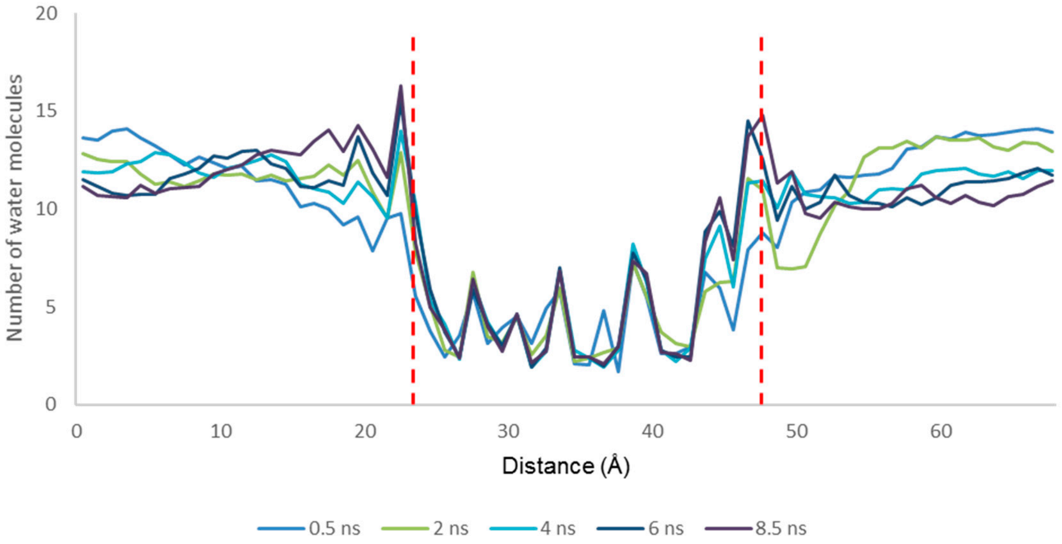

Figure S9. The concentration profile of water molecules along *z* direction for different times of MD simulations for K^+/Cs^+ system of ZM-001. The two red dashed lines indicate the location of ZM-001 surfaces in electrolyte solution.
